# The SARS coronavirus accessory protein ORF3a rescues potassium conductance in yeast

**DOI:** 10.17912/micropub.biology.001129

**Published:** 2024-03-07

**Authors:** Joel C. Rosenbaum, Anne E. Carlson

**Affiliations:** 1 Department of Biological Sciences, University of Pittsburgh, Pittsburgh, Pennsylvania, United States

## Abstract

ORF3a is an accessory protein expressed by all human pathogen coronaviruses and is the only accessory protein that strongly affects viral fitness. Its deletion reduces severity in both alpha- and beta-coronaviruses, demonstrating a conserved function across the superfamily. Initially regarded as a non-selective cation channel, ORF3a's function is now disputed. Here, we show that ORF3a from SARS, but not SARS-CoV-2, promotes potassium conductance in a yeast model system commonly used to study potassium channels. ORF3a-mediated potassium conductance is also sensitive to inhibitors, including emodin, carbamazepine, and nifedipine. This model may be used in future studies on ORF3a and related proteins.

**Figure 1. ORF3a-mediated growth effects in yeast:**
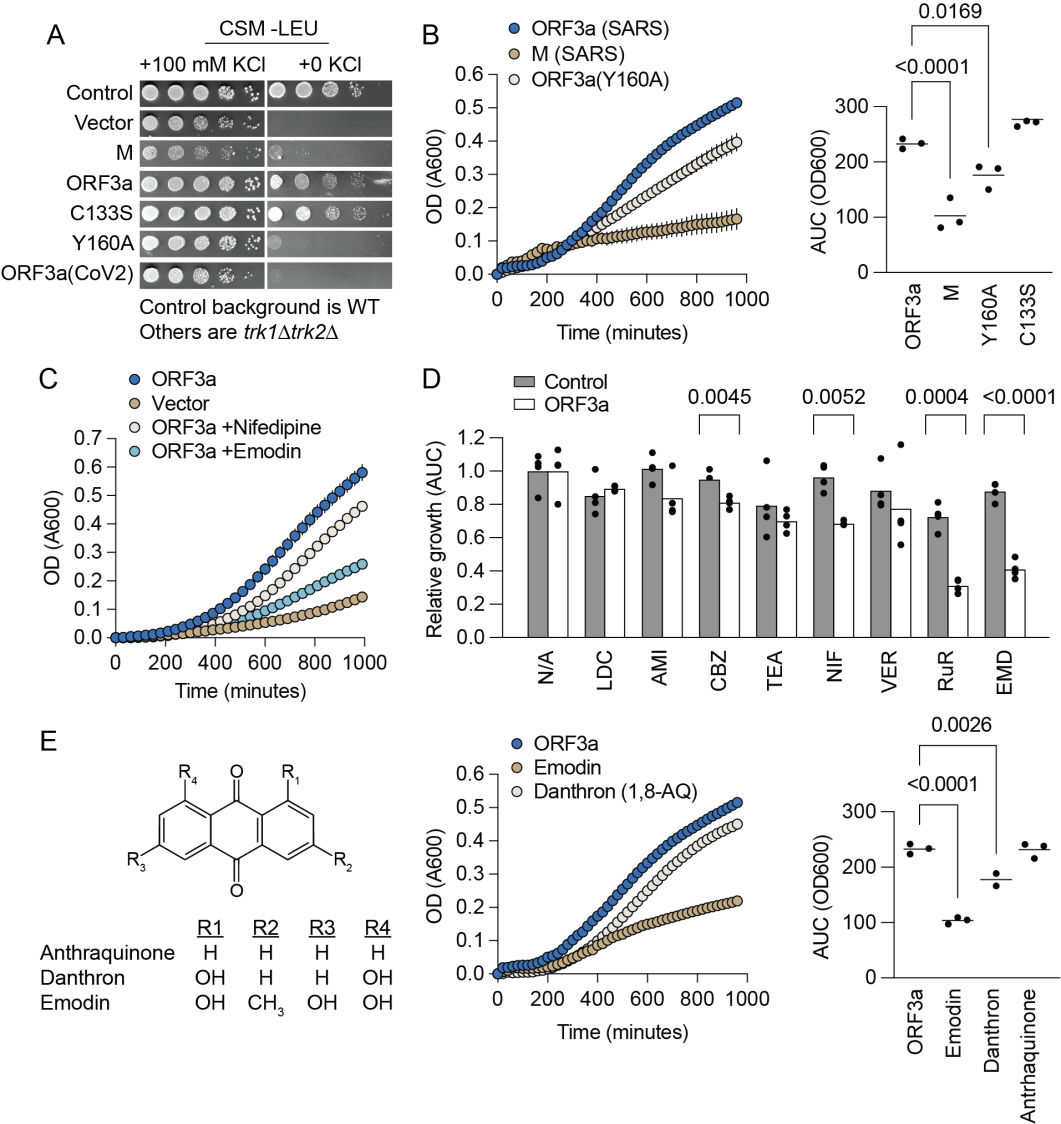
**A)** Spot test showing rescue of potassium-independent growth in *trk1Δ trk2Δ* yeast by ORF3a expression, with spots made from 10-fold serial dilutions on synthetic media (CSM) lacking leucine. **B)** Liquid culture growth assays mirror spot test results. Growth was quantified by area under the curve during a 16-hour assay. Significant differences (Dunnett’s test) are marked by brackets and annotated with *P* -values. **C)** Representative growth curve from inhibitor screening, comparing moderate (100 μM nifedipine, white) and strong (10 μM emodin, light blue) inhibition. **D)** Growth comparison in inhibitor screen, normalized to untreated controls. Data shown for wild-type (grey bars) and ORF3a-rescued *trk1Δ trk2Δ* strains (white bars). Differences were identified by Welch’s T-test marked with brackets with annotated *P* -values. E) Anthraquinone structures (left) and their impact on ORF3a-mediated growth in *trk1Δ trk2Δ* yeast (right). Brackets with *P* -values indicate significant differences (Dunnett’s test).

## Description

Coronaviruses have emerged as significant pathogens in humans over the past two decades, with outbreaks from severe acute respiratory syndrome (SARS), Middle East respiratory syndrome (MERS), and the ongoing COVID-19 pandemic (SARS-CoV-2) (Msemburi et al., 2023) . These viruses share a genome architecture that features the extended polyprotein ORF1ab, four structural proteins (S, E, M, and N), and accessory proteins named according to their position in the genome (Naqvi et al., 2020) . Of the accessory proteins, ORF3a is noteworthy for its roles in viral fitness and pathogenicity. In a mutational scanning assay, ORF3a was the only accessory protein that exhibited a strong negative selection (Bloom & Neher, 2023) . Furthermore, deletion of ORF3a attenuates virulence in SARS (Castano-Rodriguez et al., 2018), SARS-CoV-2 (Silvas et al., 2021) , feline coronavirus (FIPV) (Haijema, Volders, & Rottier, 2004) , and avian coronavirus (IBV) (van Beurden et al., 2018) , indicating that ORF3a function is essential and conserved throughout the coronavirus superfamily. Because ORF3a is required for viruses that have caused multiple epidemics in the last 20 years, studying its function could build the conceptual foundation needed to target ORF3a in future coronavirus outbreaks. ORF3a has long been considered a cation-conducting ion channel or viroporin (Lu et al., 2006) . However, recent findings challenge this classification (Miller et al., 2023) , underscoring the need for further research to clarify ORF3a's function and role in cellular processes. To address this, we have developed a yeast model system to study ORF3a in a cellular context, offering a novel approach to understanding its impact on coronavirus pathogenicity.

To investigate ORF3a function, we used a yeast strain lacking potassium transporters Trk1p and Trk2p, essential for growth in low-potassium media (Gaber, Styles, & Fink, 1988) . Heterologous expression of potassium-conducting or nonselective cation channels can rescue growth in this *trk1∆ trk2∆* background under potassium-limited conditions (Mackie & Brodsky, 2018) . This approach has demonstrated potassium conductance in ORF3a orthologs from alpha coronaviruses, such as ORF3 from the porcine epidemic diarrhea virus (PEDV) (Wang et al., 2012) .

We adapted this methodology to examine ORF3a orthologs from betacoronaviruses, a genus that includes human pathogens such as SARS, MERS, and SARS-CoV-2. We used serial dilution spot tests to assess the growth of *trk1∆ trk2∆* yeast cells expressing ORF3a from SARS under low and high potassium conditions. The structurally related M protein was used as a control (Dolan et al., 2022) . Additionally, we investigated the effects of mutations at positions C133 and Y160, sites previously established as critical for ORF3a function (Chan et al., 2009; Lu et al., 2006) . ORF3a expression rescued the potassium conductance defect in the *trk1∆ trk2∆* strains, with mutations showing distinct growth effects (Panel A). Previously, C133 was predicted to form essential disulfide bonds, but structural models did not substantiate this feature (Kern et al., 2021; Miller et al., 2023) . We found that the C133S mutation did not impair the ability of ORF3a to rescue the growth of yeast cells, further challenging the role of this cysteine in ORF3a function. In contrast, the Y160A mutation exhibited adverse effects, confirming previous findings (Chan et al., 2009) and aligning with structural models that place Y160 within the hydrophobic core of the ORF3a cytosolic domain (Kern et al., 2021; Miller et al., 2023) . Liquid culture growth assays confirmed our spot test results (Panel B). Finally, expressing ORF3a from SARS-CoV-2 produced minimal rescue (Panel A), indicating functional differences between the orthologs despite their 73% sequence identity.

Expanding on our findings, we used the liquid culture assay to evaluate how various channel inhibitors altered ORF3a-mediated potassium conductance. This included sodium channel inhibitors lidocaine (LID), amiloride (AML), and carbamazepine (CBZ); potassium channel blocker tetraethylammonium (TEA); calcium channel inhibitors nifedipine (NIF) and verapamil (VER); the nonspecific blocker ruthenium red (RuR); and the previously described ORF3a inhibitor emodin (EMD) (Schwarz, Wang, Yu, Sun, & Schwarz, 2011) (Panel C). Following normalization with untreated controls, both emodin and ruthenium red emerged as significant inhibitors of ORF3a activity in *trk1∆ trk2∆* cells, reinforcing their inhibitory potential (Kern et al., 2021; Schwarz et al., 2011) . Carbamazepine and nifedipine also displayed inhibition of ORF3a rescue. However, nifedipine was applied at concentrations (100 µM) well above its known IC50 for L-type calcium channels (0.2 µM) (Charnet, Ouadid, Richard, & Nargeot, 1987) (Panel D). The observed growth inhibitory effects in ORF3a-rescued strains suggest a direct interaction between these compounds and the ORF3a protein. This observation suggests a potential pathway for antiviral therapy exploration, although the specificity of ORF3a inhibition by emodin and other compounds requires further validation.

We expanded our investigation to include related anthraquinones to understand further the mechanism behind emodin's inhibitory effect on ORF3a-mediated rescue. We aimed to determine whether other structural analogs could replicate emodin's effects within our yeast model system (Panel E). The comparative analysis revealed that emodin's inhibitory capability was significantly greater than its analogs. Crucially, our results underscore the importance of emodin's 3-methyl and 6-hydroxyl substitutions, suggesting that these groups are critical to its interaction with ORF3a. This insight highlights emodin's potential as a promising lead in developing targeted antiviral therapies.

Our yeast assay aligns with previous research identifying ORF3a as an ion channel (Kern et al., 2021; Lu et al., 2006; Schwarz et al., 2014; Schwarz et al., 2011) , adding to the evidence supporting this characterization. Nonetheless, the indirect nature of our findings leaves room for alternative hypotheses. Recent studies suggest that ORF3a may not be an ion channel but instead works by disrupting cellular trafficking or recruiting other proteins to the cell surface (Miller et al., 2023; Yu, Sizemore, Smoot, & Perrotta, 2021) , a possibility our results do not discount. Furthermore, while ORF3a facilitates growth in yeast strains lacking native potassium transporters, this effect might be modulated by other transporters present in yeast (Bertl et al., 2003; Ko, Liang, & Gaber, 1993) . This context is crucial in interpreting the role that ORF3a plays in rescuing potassium conductance in yeast.

Notably, the comparative lack of growth rescue by ORF3a from SARS-CoV-2 in our assay suggests that the yeast rescue phenotype is sensitive to even minor structural differences in the protein. This sensitivity underscores the potential for a significant impact of the differences between ORF3a orthologs from SARS and SARS-CoV-2 on cellular trafficking and interactions. Despite these complexities, our work establishes the *trk1∆ trk2∆* yeast strain as a robust model for studying ORF3a regulation, offering a valuable tool for future research into the functions of this protein.

## Methods

**Yeast media** : We made rich medium (YPD) according to standard recipes (per liter): 10 g yeast extract, 20 g peptone, and 20 g of glucose. Complete synthetic media (CSM) according to established protocols (Bill, 2012) . These contained (per liter) 1.7 g yeast nitrogen base, 5 g ammonium sulfate, 20 g of glucose, and the following amino acids: 20 mg L-adenine hemisulfate, 20 mg L-arginine hydrochloride, 20 mg L-histidine hydrochloride, 30 mg L-isoleucine, 30 mg L-lysine hydrochloride, 20 mg L-methionine, 50 mg L-phenylalanine, 200 mg L-threonine, 20 mg L-tryptophan, 30 mg L-tyrosine, 20 mg L-uracil, 150 mg L-valine. All media were autoclaved before use. Plates were made with specified media plus 20 g of agar per liter. We supplemented media with KCl from an autoclaved 3 M stock to achieve specific ion concentrations when indicated.

**Plasmids** : For yeast expression, we used a modified pRS415 plasmid (YCp, leucine prototrophy), which contained an open reading frame under the control of the yeast constitutive TEF1 promoter (O'Donnell, Mackie, Subramanya, & Brodsky, 2017). We inserted genes of interest, including M, ORF3a, and its associated mutants, using Hi-Fi Assembly (New England Biolabs) between the XbaI and XhoI sites of the parent vector. For control experiments, we transformed yeast cells with the vector as provided.

**Yeast transformation:** Yeast strains BY4742 ( *MATα* *his3Δ1 leu2Δ0 lys2Δ0 ura3Δ0* ) and JB1512 (BY4742 strain with *trk1∆ trk2∆* ) were grown to an optical density (OD600) of 0.8 at 30°C, then centrifuged and washed twice with sterile water before transformation.

We then transformed the cells using a modified lithium acetate-based method (Gietz & Schiestl, 2007) . For each transformation, 50 µl of cells from a 5 ml culture were mixed with 100 ng of plasmid DNA and 10 µg of salmon sperm carrier DNA. These were mixed with 276 µl of PEG-TEL (40% PEG-3350, 10 mM Tris pH 8, 1 mM EDTA, and 0.1 M lithium acetate) before bringing the total volume to 376 µl with sterile water. We then transformed the cells by heat shock at 42°C for 40 minutes. Transformed cells were plated on CSM -leucine agar supplemented with 0.1 M KCl and incubated for three days at 30°C.

**Spot Test Dilution Assays:** For spot tests, strains were cultured to saturation at 30˚C in CSM -leucine supplemented with 0.1 M potassium chloride (KCl). Before assays, these cultures were diluted 1:10, and optical density measurements at 600 nm were conducted to standardize cell density. Sequential dilutions were performed in a 96-well plate format. Using a MULTI-BLOT replicator (V&P Scientific), cells were spotted onto CSM -leucine agar plates, with or without 0.1 M KCl supplementation. Plates were sealed with parafilm and incubated at 30˚C. Growth was monitored over 3 days for KCl-supplemented plates and 5 days for control plates, followed by imaging.

**Liquid Culture Growth Assays:** Strains for liquid culture growth assays were prepared similarly to those for spot tests. Growth was monitored in clear, 96-well plates, with strains diluted 1:100 into fresh CSM -leucine media containing specified KCl concentrations. Inhibitors were added as necessary. Plates were sealed with Breath-Easy sealing membranes (Diversified Biotech) and incubated in a Cytation 5 plate reader (BioTek Instruments, Inc.) at 30˚C, with optical density readings at 600 nm taken every 20 or 30 minutes over 16 hours.

**Inhibitor Screening:** For inhibitor screening, we used the following compounds at 100 µM, prepared from 1000X stocks: lidocaine (LID) in ethanol, amiloride (AML) in DMSO, carbamazepine (CBZ) in DMSO, nifedipine (NIF) in DMSO, and verapamil (VER) in water. Ruthenium red (RuR) was prepared at 100 µM from 100X stock in water. Anthraquinone, 1,8 dihydroxyanthraquinone (danthron), and emodin (EMD) were used at 10 µM from 1000X stocks in DMSO. Tetraethylammonium (TEA) was used at 10 mM from a 100X stock in ethanol.

**Statistical analysis:** Total growth was measured using the trapezoidal rule to calculate the area under the growth curve with Prism 9 (GraphPad). We assessed the homogeneity of variance among growth conditions using a Brown-Forsythe test for multiple comparisons. Following this, an ordinary one-way ANOVA was applied for overall comparison. We used Dunnett’s multiple comparisons for mean comparisons against control conditions within each data set. For our inhibitor screening experiments, growth values for each inhibitor tested were normalized against the untreated control for both the BY4742 and ORF3a-rescued JB1510 strains. Finally, the normalized growth means between wild-type and mutant strains under each treatment were statistically compared using an unpaired Welch’s T-test to accommodate unequal variances.
